# Autonomy of Children and Adolescents in Consent to Treatment: Ethical, Jurisprudential and Legal Considerations

**Published:** 2014-06

**Authors:** Alireza Parsapoor, Mohammad-Bagher Parsapoor, Nima Rezaei, Fariba Asghari

**Affiliations:** 1 Medical Ethics and History of Medicine Research Center,; 2 Faculty of Law, University of Qom, Qom, Iran; 3 Research Center for Immunodeficiencies, Children’s Medical Center and Department of Immunology, Tehran University of Medical Sciences, Tehran,

**Keywords:** Pediatrics; Autonomy; Medical Ethics; Islam; Jurisprudence; Iranian Legal System; Iran

## Abstract

Autonomy is usually considered as a main principle in making decisions about individuals’ health. Children and particularly adolescents have the capacity to take part in medical decision-making to some extent. For the most part the parent-doctor-child/adolescent triangle sides are essentially in agreement, but this may not be true in some cases, causing physicians to face problems attempting to determine their professional duties. According to Islamic jurisprudent upon reaching the age of *Taklif* (15 full lunar years for boys and 9 full lunar years for girls) no one can be treated as incompetent based on mental immaturity unless his or her insanity or mental immaturity is provend Moreover the Islamic Sharia, decrees that parents should lose their authority to make medical decisions for their children, if their bad faith or imprudence is proven, in which case a fit and proper person or an institution will be appointed to make decisions in this respect based on the child’s best interests.

## Introduction

In modern medical ethics, patient autonomy is considered a major principle in making decisions about an individual’s health, and those who receive healthcare should have the right to practice their autonomy consciously and freely; healthcare providers, on the other hand, are obligated to respect this right and allow patients to practice their autonomy in the course of their treatment^[^^1^^]^. In cases where a patient cannot exercise this right due to his or her limited ability to make medical decisions – a condition referred to as lack of capacity – a qualified person will proceed to make such decisions as the patient’s surrogate based on his or her best interests^[^^2^^]^. It should be noted that an individual’s autonomy in legitimate matters is a logical notion acknowledged by the Islamic *Fiqh*, as long as it does not jeopardize the sanctity of human life^[^^3^^]^. For this reason, according to the Paragraph 2 of Article 59 of the Islamic Penal Code of Iran, all medical and surgical procedures must be performed with the approval of the patients, their parents, guardians or legal representatives, and with due consideration for technical, scientific, and government regulations.

 In pediatrics, physicians face a wide range of intellectual and cognitive difficulties on the side of the patient concerning participation in medical decision-making; this, combined with the presence of parents, who have the right and responsibility to maintain their children according to the Article 1168 of the Civil Code of the Islamic Republic of Iran, exposes the doctor to ethical challenges while making medical decisions. These challenges are not limited to treatment alone and extend to pediatric research in particular, which we cannot afford to discuss in this paper^[^^4^^,^^5^^]^.

 The present paper aimed to examine the scope of the autonomy of children and adolescents and the extent of their parents’ authority in medical decision-making based on ethical principles and jurisprudential and legal basis. In order to make it more tangible, we have used two common cases to provide practical advices.

***Case 1: Commencing Treatment without Parents’ Consent***

A 12-year-old girl is taken to an internal medicine specialist by her teacher. Her complaints include polyuria and polydipsia, and severe weight loss. Strong suspicious to uncontrolled diabetes was made based on the history. Although the school contacted her parents many times to emphasize that she needs medical care, they ignored that as they seemed not to believe in modern medicine, and believed that herbal teas can cure her condition. The teacher asked the doctor to start proper pharmaceutical treatment and was willing to meet all the expenses.

***Case 2: Father Does Not Consent to Child’s Surgery***

A surgeon in the emergency ward had to persuade the father of a 5-year-old, in whom an acute appendicitis was diagnosed, to sign a consent form for operation. Clinical examinations and the patient’s CBC pointed to the necessity of an emergency surgery, but the father insisted on non-surgical treatment and all the surgeon’s efforts to convince him otherwise have failed.

**The Decision-Making Capacity Necessary to Exercise Patient Autonomy**

In order to exercise patients’ autonomy and preserve their integrity throughout a particular course of treatment, they need to possess the appropriate capability and decisional capacity^[^^6^^]^. In the ethical approach, decision-making capacity is a relative matter and by no means a black and white situation. A patient’s decision-making capacity can only be assessed in light of his or her specific condition, including the nature and degree of potential risks^[^^7^^]^. Assessment of decision-making capacity is influenced by the challenge between the right to autonomy on the part of the patient and principles of beneficence and non-malficence on the part of the physician^[^^8^^]^. In cases of disagreement or where the patient is not cooperating properly in the assessment process of his or her decision-making capacity, it is recommended to seek help from experts such as psychiatrists, or consultation from hospital ethics committees^[^^9^^,^^10^^]^.

 In the interaction between physician and patient, if the patient is a child and therefore not completely autonomous in making medical decisions, it is notwithstanding the physician’s duty to give the patient the opportunity to participate in the process in a manner appropriate to his or her capacity^[^^11^^]^. There have been numerous recommendations regarding the issue of pediatric patients’ participation in medical decision-making in different countries ^[^^12^^-^^14^^]^.

 Naturally, in circumstances where a pediatric patient lacks the capacity required to make a particular medical decision, it appears only logical to assign the parents the right to make medical decisions, as they are responsible for raising and maintaining their children and such responsibility entails the right to make decisions for them. On the other hand, parents’ love for their children, the responsibility they feel for their children’s life and future, and their sensitivity to their best interests, makes them the best surrogates for recognizing the pediatric patients’ best interests^[^^15^^]^.

**Children’s Age and Their Right of Decision-Making from the Medical Ethics Point of View**

Assessment of pediatric patients’ decision-making capacity should be based on their ability to evaluate their condition and the consequences of the medical decisions made, and their power to make accurate and logical deductions^[^^16^^]^. Nevertheless, it would facilitate pediatricians’ ethical decisions in assessing their patients’ capacity if the latter could be classified according to their age in such a way that patients in each group would possess similar capacity. Such classification would naturally be based on the customary assessment of the decision-making capacity of each age group; therefore in each group, a specific level of capacity can be assumed, unless proved otherwise. Ethical guidelines often recognize three stages of childhood: early childhood, middle childhood and adolescence^[^^14^^]^. In the first group, parents are basically the only decision-makers and the child is not allowed to participate in the process. In the second group, however, parents are the final decision-makers, although it is considered ethical to gain the child’s approval by offering treats and to take his or her persistent and severe resistance seriously when possible. The child’s assent is obviously sufficient in this group, and there is no need for his or her informed consent^ [^^14^^,^^16^^]^.

 The most complicated situation pertains to autonomy of adolescents before they reach full capacity. It seems adolescents’ range of capacity in medical decision-making is quite broad and may vary from complete lack thereof to perfect capacity. In this age group, it is typical to assess the patient’s capacity and base all judgments on the assessment, and physicians are ethically obliged to involve adolescent patients in medical decision-making to the extent appropriate to their capacity^[^^17^^]^.

**Children’s Age and Their Right of Decision-Making from the Point of View of Law and** ***Fiqh***

In the legal realm, certain levels of capacity are presumed for each specific age group with the turning point being the age of maturity. In an overview of the current legislations of the Western countries one can see that in the years immediately before age of maturity there are special considerations regarding a youth’s valid consent in personal matters. In Australia, the age of maturity is 18, but in case the physician determines that a patient younger than 16 is fully capable of decision-making, his or her consent is considered valid, provided that another doctor who has examined the patient prior to treatment also confirms his or her capacity in writing^[^^18^^]^. In Canada, the age of maturity is 16, although a younger patient’s consent may be considered valid under specific circumstances, where his or her physician and another independent and legally qualified medical doctor verifies the patient’s capacity and the necessity of the procedure based on the patient’s best interests^[^^19^^]^. In Ireland, the situation is more or less the same, although in the period between 16 and 18 years of age, a patient can consent to treatment, but his or her right to refuse treatment remains in doubt^[^^20^^]^. The legal age for giving consent to treatment is 16 in England, before which a patient’s consent is considered valid if his or her capacity is confirmed^[^^21^^]^. In the American legal system, the age of maturity is 18, while youths under 18 cannot make healthcare decisions without their parents’ consent^[^^22^^]^. One exception, however, is the case of emancipated minors; these are youths under 18 that have obligations similar to adults, that is, they are financially independent, are married or have children, are enlisted in the military, or have been granted the status of adulthood by a court order^[^^23^^]^. Additionally, youths under the age of 18 generally have limited rights to make certain medical decisions independently, for instance regarding treatment for sexually transmitted diseases, substance and alcohol abuse treatment, blood donation, mental health treatment and family planning services^[^^23^^]^.

 It appears that in the above-mentioned countries legislators have clearly specified the legal age for giving valid consent and at the same time have taken every measure to safeguard the right to medical decision-making for people just under the age of maturity who possess proper decision-making capacity. The responsibility to ascertain this capacity in underage patients would naturally fall to the physician.

 In the Iranian legislation and according to Shi’a *Fiqh*, stages of capacity – or competency in legal texts^[^^9^^]^ – are as follow^[^^24^^,^^25^^]^:

**1)** *Stage of Gheare Momayyez - Unawareness -:* In this stage the child has limited understanding and powers of discernment and cannot distinguish between benefit and loss, and therefore is not legally considered to have a will. The stage lasts from 2 to 7 years and corresponds to early childhood in the ethical classification.

**2)** *Stage of Momayyez – Awareness -:* This stage lies between the stage of unawareness and legal competency, and pertains to the period in which a minor is believed to have partial powers of discernment and can distinguish between benefit and loss to some extent. The law in Iran does not specify a certain age for this stage and there are no strict criteria for recognition of awareness in people as this is a faculty that can only be determined through relevant customary assessments. Based on anecdotes about Imam Ali and Imam Jafar Sadiq (PBUT), this stage may begin between ages 7 and 9^[^^26^^]^.

**3)** *Age of Taklif:* according to Shiite jurisprudents boys and girls after age of taklif are accountable for any actions they do and such they should act in accordance with God's order and avoid his prohibitions. In Iranian legislation, based on the Article 1210 of the Civil Code of the Islamic Republic of Iran and according to Shiite jurisprudents, age of Taklif is 15 full lunar years for boys and 9 full lunar years for girls^[^^27^^]^, and upon reaching this age, no one can be treated as incompetent based on mental immaturity unless his or her insanity or mental immaturity is proved.

Based on the Consistency Clause No. 30 of the Iran Supreme Court issued on December 31st, 1985, upon reaching the age of Taklif, a minor will automatically be considered competent in non-financial matters such as divorce and giving evidence in non-monetary proceedings and can begin to act independently; as regards financial matters, however, reaching age of Taklif is not sufficient grounds for competence and the minor’s mental maturity needs to be established in court as well^[^^28^^]^. Consequently, if medical decisions do not entail disposition of the minor’s property, the terms above apply and age of Taklif will be considered the criterion for the right to make medical decisions^[^^29^^]^. It is needless to say that in these cases, as in the case of adult patients, the right to make decisions is dependent upon demonstrating the necessary intellectual capacity. In other words, after reaching the age of Taklif, patients are presumed to possess the capacity for making decisions and physicians can validate their decisions and proceed with proper treatments, unless evidence is found to the contrary. Thus the child in the first above-mentioned case is legally free to make an independent decision regarding her condition, provided she possesses the required capacity to do so.

 The Shi’a *Fiqh* appears to have a similar stance toward such cases. Following an inquiry by the authors from a number of religious leaders (personal communications with Ayatollahs Safi Golpaygani, Makarem Shirazi and Moosavi Ardebili), all stated that the age criterion for possessing the capacity to give informed consent is reaching the age of Taklif as clarified by the Sharia, and neither believed there was need for any other person’s consent, parents included. In their written response, Ayatollahs Safi Golpaygani and Makarem Shirazi had also emphasized the necessity to ascertain the patient’s mental maturity and the ability to distinguish between what is in his or her interests and what is not. In other words, they were of the opinion that it is necessary for the physician to establish the patient’s capacity for medical decision-making. Some contemporary Faqihs believe that if a patient is of age, but is not mentally mature and therefore lacks the decision-making capacity, his or her parents’ consent is required in addition to the patient’s permission or assent^[^^30^^]^.

1. *Stage of Maturity – Roshd-:* Adulthood is the stage when a patient’s capacity and competence in all legal financial or non-financial matters is presumed, and supervision or consent of parents in those matters is seemingly unnecessary.

According to the Article 1209 of the Civil Code of the Islamic Republic of Iran prior to the 1982 reforms, a person was considered legally mature upon reaching 18 full solar years and his or her parents’ guardianship would expire with no court proceedings. After the Civil Code reforms the age specification has been repealed, although anyone 18 years and older is considered independent and accountable for his/her actions and decisions as common legal practice^[^^31^^,^^32^^]^.

 Based on the facts stated above, it can be inferred that according to the Iranian legal system, in cases where a form of payment is not required for medical treatment, reaching the age of Taklif indicates a patient’s independence in making medical decisions, unless he or she is proven to lack capacity. However, if the patient needs to have access to his or her property in order to pay for the treatment, he or she needs to have reached the age of 18.

**The Challenges a Physician Faces Regarding Determination of a Patient’s Capacity**

In Iran, according to the law and *Fiqh*, patients have the right to make medical decisions, if their decision-making capacity is established, or in terms of *Fiqh*, they should have the ability to distinguish between what is in their interests and what is not. The fact that for girls the age of Taklif, after which a person is considered competent, is lower raises concern about their mental capacity to make decisions in the years immediately following the age of Taklif as clarified by the Sharia.

 Unwary acceptance of the Taklif age as the criterion for young patients’ right to make medical decisions regardless of the sensitivity of such decisions can sometimes cause problems for doctors and the healthcare system. Considering that making medical decisions bears directly or indirectly upon the patients’ control over their body, which is no less significant than their control over their finances, it seems essential to raise the age of decision making capacity, particularly in case of young girls. As the author of the acclaimed book on *Fiqh*, entitled *Orvatolvosqa* states, in marriage, which is a non-financial matter like medical treatment, a young girl’s consent is necessary in addition to that of her parent only when apart from being of age, she has reached the stage of mental maturity necessary to make an independent decision in this respect. Otherwise she can only get married with the guidance and supervision of her parent and by his permission^[^^33^^]^. This point along with some other considerations caused the legal age for girls to be married to be raised from 9 to 13^[^^34^^]^. It seems that in medical decision-making likewise there should be no discrimination between girls and boys with regard to the age of decision making capacity; it is therefore recommended that the age requirement for the right to make medical decisions be raised to 15 for girls as well, and the laws regarding decision-making capacity be duly reformed. 

 On the other hand, it should be noted that emotional and responsible nature of the Iranian family does not allow parents to be indifferent to their young children’s fate, especially their daughters, even though they may have reached the age of maturity and possess the decision-making capacity. Even if the reform suggested above regarding the age requirement for girls to make medical decisions is enacted, the significant role of the young patient’s family cannot be overlooked. Proper use of communication skills in interactions with patients and their families seems to be essential in order to reach medical decisions that not only ensure individuals’ right to autonomy, but also produce the best results with the least amount of anxiety and stress for young patients and their families.

**Ethical Solutions for Parents’ Wrong Decisions regarding their Children**

In the course of treatment, physicians may come across instances of wrong decisions made by parents that are clearly not in the child’s best interests. Deliberations on the reasons why parents are selected as surrogates in medical decision-making highlight two basic presumptions in this respect that parents are responsible and caring, when it comes to their children, and that they have their children’s best interests in heart. If the two above-mentioned presumptions no longer apply, it does not seem morally appropriate that parents continue to enjoy their right to surrogacy^[^^14^^,^^35^^]^. There are inevitably disagree-ments as to what the exact criteria for determination of a child’s best interests may be, but one valuable measure for pediatricians could be the principle of protecting children against serious harm, pain and death^[^^36^^,^^37^^]^. In guidelines offered in medical ethics literature there are restrictions on the scope of autonomy of adult patients in medical decision-making, for instance a patient’s wish to commit suicide is invalid; likewise, multiple restrictions apply to the autonomy of parents regarding their children^[^^38^^]^. For example, in most countries people have the right to refuse life-saving treatments, while no one is granted this same right regarding their children^[^^15^^]^. Similarly, parents cannot refuse their children life-sustaining treatments on account of their own religious beliefs^[^^36^^]^. Parents are morally obliged to make medical decisions based on their children’s best interests, not their own wishes and well-being, and if the physician decides that their decision is not in the best interests of the child, a reliable authority, that is, the court or ethical committee, can preserve the child’s rights in an unbiased manner.

 In situations like the second case above, where the parents’ refusal or consent to a certain treatment is clearly in conflict with the child’s best interests, the doctor should offer adequate explanations and make the parents aware of the consequences of their decision so they can eventually reach an agreement^[^^11^^]^. In case the physician does not succeed in changing the parents’ mind, the situation can be resolved by referring the matter to the ethical committee. In rare occasions, where child abuse or neglect is suspected, it might be necessary to take the matter to court^[^^15^^]^.

 These approaches apply only if the child is not in an emergency or a life-threatening situation. In emergent conditions, the physician can disregard parents’ refusal and proceed with urgent medical intervention until the situation is no longer life-threatening^[^^21^^]^. 

 From the point of view of *Fiqh*, parents or legal guardians are obligated to have only the child’s best interests in mind and not reject measures upon which the child’s life depends within reasonable limits, as this is the responsibility they have been charged with^[^^29^^,^^39^^]^.

 Such ethical views on the scope of parents’ rights to make decisions regarding their children appear to be acceptable in the legal system of Iran. According to the Article 1184 of the Civil Code of the Islamic Republic of Iran “if the natural guardian of a child is unmindful of his ward’s well-being and manages his or her property in a manner that brings about loss, the court will, on application by the relatives of the child or by request of the Public Prosecutor, and after the establishment of the incapacity or dishonesty of the guardian, discharge the guardian of his duties and conclude his management of the child’s properties, and will appoint a proper financial trustee in his place.” This article may appear to be related to children’s properties and financial affairs, but can extend to more important matters by the same token.

 Moreover, based on the Article 1173 of the Civil Code of the Islamic Republic of Iran, “If the physical health or moral education of a child is endangered as a result of carelessness or moral degradation of the father or mother who have custody of the child, the court can take any decision it deems appropriate regarding custody of the child upon request of his or her relatives, guardian or the Public Prosecutor.”

 Such laws indicate that custody is contingent upon preservation of the child’s well-being or, in other words, best interests. 

 It is noteworthy that in the Shi’a *Fiqh* the judge can dismiss a parent’s guardianship of his child if his incapacity or dishonesty is established. The author of the book *Javaherulkalaam* who is also a renowned expert on the Shi’a *Fiqh* maintains that even if evidence and circumstances indicate that the father or paternal grandfather of a minor or an insane person has caused them to incur material loss, the judge needs to take away their control over their ward’s property^[^^27^^]^. It should be noted that in cases like this, called non-litigious cases, the court should start proceedings directly even if there is no dispute or complaint^[^^40^^]^.

 In response to our inquiry regarding whether it is permissible to perform a necessary but not urgent procedure on a minor, examples varying from administration of antibiotics to surgeries, most responses (personal communications with Ayatollahs Safi Golpaygani, Makarem Shirazi and Moosavi Ardebili) pointed to the necessity of seeking parents’ consent, although they considered it acceptable to disregard the parents’ refusal to consent to procedures in case of life-threatening situations.

## Conclusion

One of the most important missions of medical ethics is to protect the rights of all individuals and ensure that they exercise autonomy within their intellectual ability and capacity. According to the Shi’a *Fiqh* and Iran’s written laws, the age requirement for the right to make medical decisions is reaching the age of Taklif, which is 15 full lunar years for boys and 9 full lunar years for girls. This sex-based difference in age of decision making capacity seems to be in discordance with the current medical ethics guidelines and is therefore unjustifiable. The concern is mostly regarding young girls’ vulnerability due to the fact that they can practice their medical decision-making right at an earlier age. In order to exercise this right, an adolescent’s capacity needs to be established, as is the case with adults; as regards young girls who have just come of age, more care needs to be taken in establishing their capacity.

 Physicians, health policy makers and legislators should reach an agreement regarding the age of capacity for medical decision-making based on their estimations of the average age for attaining capacity in the general public and the society’s overall welfare, as has been done in case of age of maturity for getting married and taking control over one’s property. The authors do not believe there should be any discrimination between the sexes in this regard.

 In many cases, it is crucial that medical decisions be made in the shortest time possible so that treatment can begin immediately. A physician’s determination of an adult patient’s capacity puts him or her in a complicated situation, particularly if the patient disagrees with the doctor or his or her parents. Under these circumstances, physicians are supposedly authorized to proceed with life-sustaining measures, but the moral and legal liabilities of such decisions are substantial and therefore stressful for doctors, especially if they occur frequently over time. Moreover, complexities of these decisions oftentimes necessitate exchange of views among various people. 

 It seems that it would be helpful for health centers to have access to an easily reachable committee charged with the responsibility to make such decisions. Such a committee should be endorsed by the nation’s legal system and be answerable to any possible grievances. On account of the authority bestowed by *Fiqh* and the law on physicians regarding distinguishing lifethreatening situations and medical emergencies, and determination of an adult patient’s mental maturity, such a committee can ensure patients’ best interests with the lowest mental, emotional and legal consequences for doctors.

## Authors’ Contribution

A. Parsapoor: Concept, Acquisition of data, Interpretation, Drafting of the manuscript

M. Bagher Parsapoor: concept, critical revision of the manuscript and approval of the article. 

N. Rezaei: concept and approval of the article. 

F. Asghari: critical revision of the manuscript and approval of the article.

All authors approved the final version of the article.
